# Eye, Ear, Nose, and Throat

**Published:** 1895-07

**Authors:** 


### *SELECTIONS AND ABSTRACTS.*

### EYE, EAR, NOSE, AND THROAT.

Edited by Dunbar Roy, A.B., M.D.

The meeting of the American Medical Association in Baltimore was a notable one in many respects. First and foremost was the large attendance of the Association as a whole, and the second was the unusually large attendance in the various sections. Then again the papers and discussions were most apt and excellent. The Section of Ophthalmology was one of the most largely attended of any section, so much so that extra seating capacity had to be made on the second day. The *personnel* of this section could hardly have been improved since there were men present from all sections of the Union, and men who by reputation are recognized leaders in ophthalmological work in that section of the country from which they come. Dr. Jackson of Philadelphia, and Dr. Würdemann of Milwaukee, made most excellent officers. There was a "snap" about the section work which was especially noticeable, due in a large measure to the able management of the presiding officer. The papers were all good and held within the limit of the time.

The Section on Otology and Laryngology, while not so largely attended as the one on Ophthalmology, made up this deficiency, however, in the excellency of the papers read. It was deeply regretted by the section that Dr. Gallaher, the secretary, could not be present, but in its president, Dr. Fulton of St. Paul, the section found a most excellent presiding officer. This was the first year that the section decided to have its transactions published in book form, and those who may wish to subscribe will find its contents of great practical value. The discussion on the treatment of antrum disease was probably the most interesting and animated during the whole meeting. In both of those sections, I am sorry to say that, numerically, the South was very poorly represented. This should not have been the case, since in the South we have many most excellent men in this special line of work who could have ably represented this section of the country in articles before this body. We earnestly trust that at the next meeting, which convenes in Atlanta, we may have a large representation of Southern ophthalmologists and otologists.

---

### The Rational Treatment of Acute Otitis Media.

An elaborate study of the treatment of acute inflammation of the middle ear from both a clinical and bacteriological standpoint is undertaken by Gradenigo and Pes in the *Archiv. f. Ohrenkeild,* xxxviii, 1 and 2.

Gradenigo's aim is to find a method of treatment based upon the facts we have learned from bacteriology, and one which at the same time conforms to the well established rules of present-day surgery. Some idea may be gained of the care which has been taken in the preparation of the article from the fact that there are 142 references to the literature of the subject. The historical sketch is sufficiently complete, and the authorities carefully ranged in favor of or against the various methods in vogue. Treatment of the nose and naso-pharynx, poultices, hot and cold water applications (of which the most extraordinary is De Rossi's instillations of ice water), the air douche and catheter, the stage of the disease in which paracentesis is indicated, the many antiseptics and astringents are all discussed, and the point made that the only things agreed upon are paracentesis and asepsis.

Gradenigo thinks it of the utmost importance from a therapeutic standpoint to study the development and progress of the disease. It is known that acute otitis media can be caused by any one of the several micro-organisms; of these comes first the diplococcus of Fraenkel. Nevertheless accessory causes are not to be overlooked, especially chronic catarrhal conditions; the divisions of middle ear inflammation with the catarrhal and purulent cannot be admitted from the bacteriological standpoint. The development of an acute process depends largely upon factors apart from micro-organisms. Netter found in the ears of young children the same microbes which are present in acute middle ear inflammation in the adult. Further observations in this direction are necessary. If we except cases in which there is a traumatic lesion of the drumhead, we may conclude that all infections of the middle ear are secondary and that the micro-organisms enter the middle ear by the Eustachian tubes. Gradenigo believes that infection takes place in this way even in typhus, measles, scarlet fever, pneumonia, etc., in all of which the general condition of the patient and the resisting power of the mucous membrane are greatly lowered. While over 100 species of micro-organisms have been described as existing in the naso-pharynx, the sort of germ is obviously of much more moment than the number of kinds present. It is further known that many pathogenic micro-organisms do not possess under all circumstances sufficient virulence to cause an acute infection, but are apparently influenced by chemico-physical conditions with which we are not sufficiently familiar. It is therefore impossible to predict for any particular micro-organism any certain course. Furthermore, although but little remains to be learned as to the kind of germ which can set up an acute inflammation, no one has up to the present made bacteriological central examination of the various methods of treatment. This the authors have endeavored to do.

When the infectious character of the disease was known, trials were made with antiseptic solutions. These were unsuccessful, because the quantity of the weaker strength which could be used was relatively too small, and stronger solutions would cause so much irritation as to aggravate the process; and even if one could use enough of the stronger solution the folds and thickness of the mucous membrane and irregularities of surface in the middle ear make thorough asepsis impossible. One can observe also that the inflammatory process in the middle ear, as in other organs, has a tendency to pursue a certain cycle. An adequate knowledge of this course is necessary to the correct treatment.

Another question which arises in many cases is paracentesis. It is, of course, possible to change by means of this operation a catarrhal into a purulent inflammation if proper antiseptic precautions are not observed, and it may even be a dangerous operation if, through uncleanliness or irrational inflations a secondary infection occurs.

While Gradenigo considers the air douche injurious, he thinks the use of antiseptic irrigations equally pernicious. The increase of the pain follows the washing at a certain interval, and for this reason is frequently not ascribed to it, especially if the washing out of the ear is practiced often. Washings are recommended to prevent a collection of pus in the external meatus. The aim is better attained by drainage. The washings may, in some who have sensitive skins, cause eczema, furuncle, etc. Even more may properly be urged against irrigation *per tubam.*

Against too rapid conclusions as to the therapeutic worth of any particular treatment, the authors urge the fact that acute inflammations of the middle ear last a very variable time. However, in adults with scars which are easily broken through, spontaneous perforations occur early, and as a rule recovery takes place in a few days. With chronic catarrh and thickened drumheads severe complications frequently occur, while in the aged in whom the inflammatory process is not so acute and in whom the exudation remains in the middle ear for several months, marked deafness and severe subjective symptoms result.

Gradenigo's method of treatment is as follows: When the acute otitis is in the first stage, the pain having lasted but a few hours, or having moderated when only the upper segment of the drumhead is reddened and hearing power on the affected side is still good, an abortive treatment should be instituted, i. e., rest in one's room or bed, light diet, gargles, washing of nose with lukewarm salt-water, and instillation in the ear of 1.5 to 2 per cent. solution of carbolic acid. The use of a solution in glycerin is, in the author's opinion, irrational. Carbolic acid in glycerin or in oil has a very weak antiseptic action. The watery solutions work more energetically; to prevent maceration of epithelium an 0.8 per cent. salt solution is added.

If the abortive treatment fails, an early vertical paracentesis in the posterior segment of the drumhead is made. Before the operation, the auricle and external meatus are to be washed with a lukewarm 1:1000 sublimate solution and a little of a 10 per cent. cocaine solution in 1 per cent. carbolic acid solution dropped in the ear. After paracentesis, and whether exudation occurs or not, neither washing nor other irritating operation is done, because if exudation is present it will shortly find its way out, and if none be present the artificial perforation will close in twenty-four hours without complicating matters. If the perforation does not appear to be large enough a second incision is made which crosses the lower end of the first at right angles. The pain is much lessened by the use of cocaine. While exudation takes place the perforation is to be kept open and all washings are to be avoided. To prevent the too early closure of the perforation, drainage is to be secured thus: After perforation the secretion and blood in the meatus are carefully removed by means of a tampon, so used that the drumhead is not touched. Only when the secretion is very great is a lukewarm 1:10000 sublimate solution gently used. Then with forceps, under aseptic precautions, and using the eye and speculum, a thin strip of iodoform gauze is deeply inserted in the external canal; the end of the gauze is, however, not allowed to touch the drumhead, which it would irritate. The correct insertion of the tampon is of great importance, since if too deeply inserted it cannot be borne, and if not inserted far enough it will not prevent the pus collection in the deeper parts of the meatus. The strip of gauze makes possible a capillary drainage. At the outer end a few layers of iodoform gauze are placed, and if the secretion is very abundant, absorbent cotton gauze may be applied and a bandage put on. If necessary the dressing may be applied twice a day. Sometimes the absorbed cotton may be attached to the auricle with collodion. When, after two or three weeks, the acute symptoms have disappeared but pus is still present, an ear bath, lasting fifteen or twenty minutes, of a 1:10000 sublimate solution should precede the dressing—of course a large opening in the drumhead must exist. The air douche was very rarely used, and Gradenigo believed that it not infrequently caused a recurrence of the acute symptoms, especially in children.—*Annals of Ophthalmology and Otology*.

